# A Novel Energy Harvester for Powering Small UAVs: Performance Analysis, Model Validation and Flight Results

**DOI:** 10.3390/s19081771

**Published:** 2019-04-13

**Authors:** Rocco Citroni, Franco Di Paolo, Patrizia Livreri

**Affiliations:** 1 Department of Engineering, University of Rome Tor Vergata, 00100 Roma, Italy; franco.di.paolo@uniroma2.it; 2 Department of Engineering, University of Palermo, 90128 Palermo, Italy; patrizia.livreri@unipa.it

**Keywords:** energy harvester, MAV, power consumption model, nano-antennas, dipole rectenna array, perpetual flight

## Abstract

The proposed work aims at exploring and developing new strategies to extend mission parameters (measured as travel distance and mission duration (MD)) of a new class of unmanned vehicles, named Micro Air Vehicles (MAVs). In this paper, a new analytical model, identifying all factors, which determine the MAV power consumption, is presented. Starting from the new model, the design of a nanoarray energy harvester, based on plasmonics nano-antenna technology is proposed. The preliminary study was based on a 22,066,058 22,066,058 × 62,800-dipole rectenna array producing an output power level of 84.14 mW, and an energy value of 2572 J under a power density of 100 mW/cm² and a resonant frequency of 350 THz as input conditions. The preliminary analytical results show a possible recharge of an ultra-fast rechargeable battery on board of a MAV and an MD improvement of 16.30 min.

## 1. Introduction

Considered as the eyes and ears behind the enemy lines, today Unmanned Air Vehicles (UAVs) are playing an important role in global society due to their ability to carry a payload and to their compact dimensions. These air vehicles can operate and monitor complex scenarios where the presence of onboard human pilots is unnecessary [1]. Today, thanks to the miniaturization and compact dimensions of the electronics components, a new class of unmanned vehicles, named micro air vehicles (MAVs), hand-launched (their weight being less than 100 g) and powered by electric engines, is emerging. Currently, MAVs can operate where larger UAVs cannot, such as inaccessible and contaminated areas. Despite some benefits, several technological limits must be overcome. Typically, the mission parameters of these vehicles are limited to a few meters and minutes. The loss of a MAV’s performance is due to energy restrictions of the battery on board. Considering a large area to be monitored as fast as possible, with the current technology, a single drone will be not able to land, recharge the battery and take off again in a few minutes. Therefore, to make this unmanned technology more attractive it is mandatory to find a solution for the problem of how to increase the mission parameters [2,3,4]. The authors suggest two directions, which are extensively detailed below.

First, a model identifying all factors that determine the power consumption of a drone with a negative impact on mission parameters is developed and implemented. Starting from the proposed model, mission parameters can be increased with an energy harvesting (EH) technique. This technique is capable of extending the mission parameters considering a renewable, intermittent source of energy [3,5,6,7,8,9,10]. The Sun is the most important renewable energy source to generate the electricity necessary for any human activity. The Sun radiates energy mainly in the form of visible light, and infrared radiation with small amounts of ultraviolet [11,12]. 

Solar radiation is usually considered as shortwave infrared (SWIR), radiation typically classified as belonging to the 0.7 to 2.5 μm wavelength range [13]. The wavelengths in the visible region are currently harvested using solar cells based on the photovoltaic (PV) effect. However, this technology has several problems for unmanned applications. Quantum technologies are strongly dependent on daylight, which makes them also sensitive to weather conditions. Only photons charged with an energy equal to the silicon bandgap can be harvested efficiently. This effect decreases the solar cell efficiency to only 21% [11]. A low-cost alternative to PV effect is a novel harvester constituted by nanosized rectennas (submicron antennas combined with ultra-high frequency diode rectification) based on plasmonics technology [14,15]. This technology would theoretically allow a more efficient, direct conversion of electromagnetic radiation (visible light frequency range) into electricity, above the Shockley and Queisser (S&Q) limit. In contrast to solar cells, which are limited by material bandgaps, this technology is based on electromagnetic waves that induce a time-changing current at the same frequency of the wave incident on the antenna surface. This time-changing current can be rectified in order to produce a direct current (DC) power and to power supply an external load. For this reason, an ultra-high speed rectifier based on the tunnel effect placed at the feed point of a nano-antenna is mandatory [16,17]. Therefore, compared to quantum technology, this novel harvester, which can be seen as a small nano-generator, represents an alternative way to supply power to a MAV for longer times, even when the Sun is heavily shaded. These new devices, thanks to the development of new techniques, such as electron beam lithography and atomic layer deposition (ALD), are able to assure the level of miniaturization required for our purposes [16]. This significantly reduces the size and weight of the battery, thus improving the mission parameters. This paper is arranged as follows: Section 2 presents a model based on a commercial quadcopter identifying all factors that determine the power consumption of a drone with a negative impact on mission parameters. Section 3 describes the design and simulations of the novel harvester based on plasmonics technology. The harvester circuit impedance has to be matched with the load (a typical DC/DC converter for harvesting applications) to optimize the power transfer. Therefore, in this section, an optimal matching between harvester and load is proposed simply by tuning the impedance of the harvester with that of the load. The harvested energy stored in a buffer is described in Section 4. Concluding remarks are given at the end of Section 5.

## 2. Several Scenarios Proposed for Electric Power Consumption Model

In this section, in order to determine the thrust and the power consumption of the MAV, the weight of each component must be estimated. Subsequently, a novel model based on a commercial quadcopter able to determine the power consumption of a drone with a negative impact on mission requirements is proposed. The numerical analysis for this model has been carried out through the software Mathcad R2014 with an academic license.

### 2.1. MAV Platform Design

In order to determine the thrust and the power consumption of the MAV, the weight of each component must be estimated. Table 1 shows the total weight of the MAV and all its components [3].

### 2.2. A Power Consumption Model for MAVs

This more sophisticated model identifies all factors that determine the power consumption of a small drone with a negative impact on mission parameters (MD and travel distance) considering various flight scenarios. MD of a MAV is a function of the power P: MD = f (1/P) therefore, to maximize MD, it is necessary to minimize the power consumption. This situation triggers the need for strategies to reduce the power demand onboard drones. An important need is the ability to understand the way in which power is used and spent. In this way, it is possible to identify what are the factors (maneuvers, movements and flight profile) that can involve more energy investment, and therefore faster battery discharge. The factors that determine the power consumption of a drone can be grouped as [3,18]:

(1) Impact of Motion: The motions of a drone can be divided into hovering, vertical and horizontal moving. This paper investigates the power consumption of a drone in each motion type.
(2) Impact of Weight: We study how different weights of payloads limit their travel distance.
(3) Impact of Wind: The major environmental factor that affects the drone is wind. We study the power consumption of a drone in headwind conditions.

#### 2.2.1. Impact of Motion: Hovering Condition

In this paper, we are considering a quadcopter as a drone. A quadcopter produces both lift and thrust by using one or more rotors. In our first case, we have a hovering condition where the thrust of the rotor is used to completely equilibrate gravity. The thrust *T* produced by the rotor is [19,20,21]:

(1) $$T=2\rho A\bar{V}w$$

where *ρ* = 1.225 kg/m^2^ is the air density, *A* is the area of the MAV (0.033 m²), $\bar{V}$ is the resultant velocity and w is the induced velocity induced by rotor blades:

(2) $$\bar{V}=\sqrt{{\left(w-V\sin\alpha \right)}^{2}+{\left(V\cos\alpha \right)}^{2}}$$

where *V* is the speed of the object relative to the fluid, α is equal to the angle-of-attack of the rotor plane. Now, considering only the hovering condition *V* = 0, so that $\bar{V}=w$, and using momentum theory:

(3) $$T=2\rho Aw^{2}$$

In hovering condition, the net thrust *T* of the four rotors pushing the drone up must be equal to the gravitational force $W_{tot}=m_{tot}g$ pulling it down:

(4) $$m_{tot}g=2\rho Aw^{2}$$

which delivers an expression for the induced velocity:

(5) $$w=\sqrt{\frac{m_{tot}g}{2\rho A}}$$

The total airplane mass $m_{tot}$ in Equation (5) is represented by:

(6) $$m_{tot}=m_{bat}+m_{struct}+m_{prop}+m_{harvester}+m_{av}+m_{pld}$$

where battery, structure, propulsion, harvester, avionics and payload masses, have to be optimized. The power needed for the propulsion is given by:

(7) P=DV+Tw

where D is the drag, V=0 (hovering condition) and $T=m_{tot}g$, so Equation (7) becomes:

(8) $$P=Tw=\sqrt{\frac{m^{3}{}_{tot}g^{3}}{2\rho A}}$$

**Figure 1:**
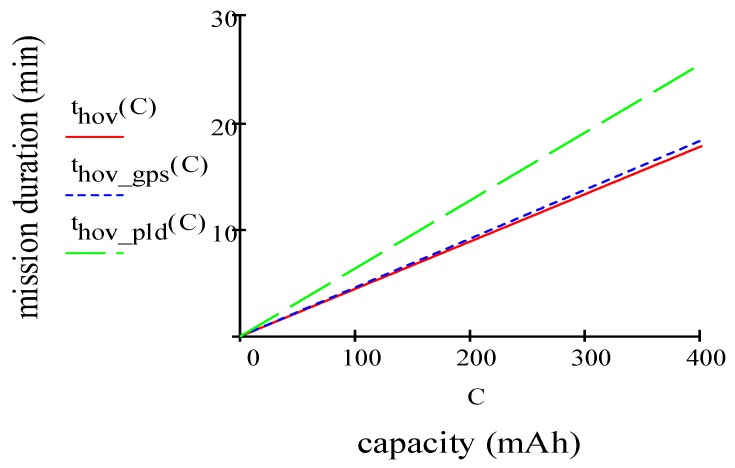
Mission duration vs capacity.

Therefore, to reduce the ideal power, we need to decrease weight, increase the rotor blade size or simply fly at a lower altitude with the capabilities to operate with environmental temperatures. The next step is that of calculating the thrust needed to lift and hover the quadcopter. As rule suggests, thrust should be more than twice the weight of the quadcopter. Total thrust T will be T=2.0×80=160 g. Now, in order to calculate the thrust action on each motor, it is necessary to divide the total thrust T for the number of motors =160/4=40 g. Maximum MD in hovering condition can be written as:

(9) $$t_{hov}=\frac{E_{\max}}{P_{tot}}\cdot \eta =\eta \cdot \left(\frac{60}{1000}\right)\left(\frac{C\cdot V_{n}}{p_{tot}}\right)$$

where efficiency η= 0.80 includes propeller and motor, $\frac{60}{1000}$ converts mAh into Amin, C is the battery capacity (in mAh), $V_{n}$ is the nominal battery voltage (in Volt) and $p_{tot}$ represents propulsion (taking four motors into account), payload and avionics power consumption. For this quadcopter, we are considering a Li-Po battery with an operating voltage of 3.7 V and a capacity of 400 mAh. By taking into account Equations (5)–(9), the results are w= 1.73 m/s, $p_{prop}=$ 1.11 W (calculated for four motors), $p_{av}=$ 1.57 W, $p_{pld}=$ 1.21 W, $p_{tot}=p_{prop}+p_{pld}+p_{av}=$ 4.00 W, $p_{tot}=$ 3.88 W without GPS, *p_tot_* = 2.79 W without payload. By using Equation (9) we have *t_hov_* = 18.16 min, $t_{hov}=$ 18.30 min without GPS, $t_{hov}=$ 25.46 min without payload. Figure 1 plots mission duration (MD) in min vs. capacity in mAh. In several conditions as hovering, hovering without GPS and hovering without payload, MD increases according to the battery capacity. However, as the capacity of battery extends, the increase in MD becomes ineffective.

From the results, we observe that the drone can maintain a sufficiently steady flying altitude with steady power consumption.

#### 2.2.2. Impact of Motion: Vertical Condition

**Figure 2:**
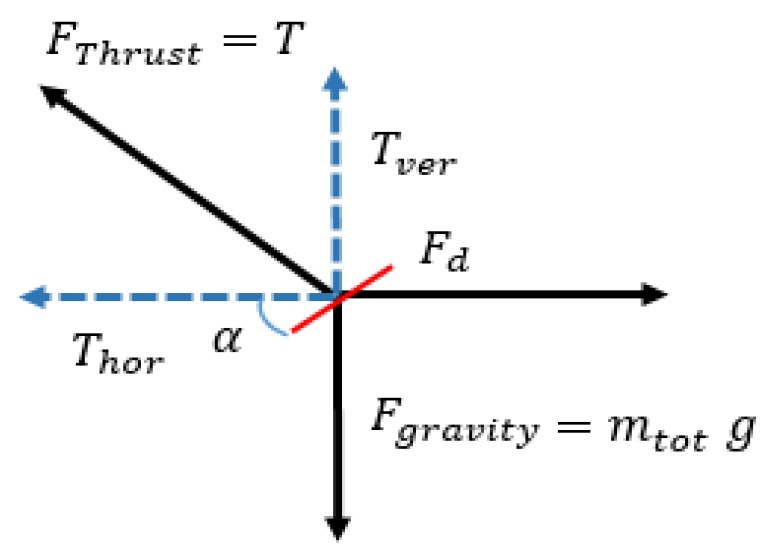
Forces on the MAV during the flight.

When considering a vertical condition, the MAV rises and falls quickly. This happens if the thrust of the four MAV rotors is greater than the mass and gravity force. Figure 2 shows the forces on the MAV during the flight [18,22,23,24,25].

(10) $$F_{Thrust}-F_{Gravity}-F_{d}=m\dot{v}$$

$F_{Thrust}=T\;,$ represents the thrust force, *F_Gravity_* represents the gravity force, $F_{d}$ represents the aerodynamic drag, *m* is the mass of MAV and *ν* is the speed of the object related to the fluid. *F_d_* can be written as:

(11) $$F_{d}=\frac{1}{2}\rho Av^{2}C_{D}$$

where: *ρ* = air density (kg/m^3^); *A* = top area of the MAV; *ν* = speed of the object related to the fluid (m/s); *C_D_* = drag coefficient (dimensionless constant fixed at 0.80 in this case study). When replacing the values, Equation (10) can be rewritten as:

(12) $$T-m_{tot}g-\frac{\rho }{2}c_{D}A_{eff}v^{2}=m_{tot}\dot{v}\;$$

where: *T* = total motor thrust (N); *g* = gravity of Earth (9.81 m/s^2^). Equation (12) can be rewritten as:

(13) $$\frac{T}{m_{tot}}-g-\frac{\rho }{2m_{tot}}c_{D}A_{eff}v^{2}=0$$

From (13) we get the maximum climbing rate (vertical speed):

(14) $$v_{ver}=\sqrt{2\frac{T-m_{tot}g}{\rho c_{D}A}}$$

The thrust to weight ratio $\mathrm{TWR}=T/W=T/m_{tot}g$ is the main dynamic characteristic that will determine the drone flight profile. To take off a MAV needs *TWR* > 1 so it has a net acceleration upwards. When hovering, thrust is purely vertical, as the MAV pitches forward, the thrust acts at an angle named angle of attack *α* shown in Figure 2. The angled thrust is made up of a vertical and horizontal component. The last component acts on the MAV so as to move it forward. This requires the *TWR* to be at least 1.3 for an approximate maximum angle of attack of 40 degrees. When introducing the thrust over weight ratio in Equation (14) we get the climbing rate as a function of the thrust-weight ratio *TWR*:

(15) $$v_{ver}=\sqrt{\frac{2m_{tot}g}{\rho c_{D}A}}\cdot \sqrt{\left(TWR-1\right)}$$

The thrust weight ratio has to be greater than 2. Beside the thrust weight ratio, the maximum climbing rate is a function of the weight ($m_{tot}g$) and the size (area *A*) of the quadcopter, as well as the aerodynamic drag coefficient $C_{D}$. The power consumption of the propulsion motor will be:

(16) $$p_{prop}=Tv_{ver}=T\sqrt{\frac{2m_{tot}g}{\rho c_{D}A}}\cdot \sqrt{\left(TWR-1\right)}$$

**Figure 3:**
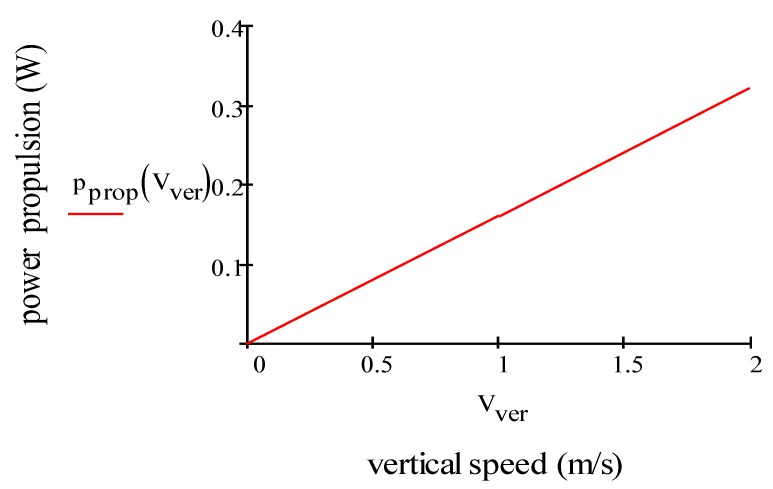
Electric power consumption for the propulsion of one motor vs. vertical speed.

With Equations (14)–(16), we have the results $v_{ver}=$ 2.24 m/s, $p_{prop}=$ 1.43 W (calculated for four motors), $p_{av}=$ 1.57 W, $p_{pld}=$ 1.21 W, $p_{tot}=p_{prop}+p_{pld}+p_{av}=$ 4.21 W. By using Equation (9) $t_{ver}=$ 17.20 min. Figure 3 plots power propulsion (*W*) vs. vertical speed (m/s) of one motor. Electric power consumption of the propulsion motor increases slightly and linearly when the drone ascends steadily.

#### 2.2.3. Impact of Motion: Horizontal Condition

In this condition, the drone moves horizontally without altering neither its altitude nor its cruising velocity. In forward flight condition, Equation (12) can be rewritten as [18,22,23,24,25]:

(17) $$\sqrt{1-{\left(\frac{m_{\mathrm{tot}}g}{T}\right)}^{2}}\cdot T-\frac{\rho }{2}c_{D}A_{\mathrm{eff}}v^{2}=m_{\mathrm{tot}}v$$

due to the air drag, the quadcopter will reach a speed limit without further acceleration:

(18) $$\sqrt{1-{\left(\frac{m_{\mathrm{tot}}g}{T}\right)}^{2}}\cdot \frac{T}{m_{\mathrm{tot}}}-\frac{\rho }{2m_{\mathrm{tot}}}c_{D}A_{\mathrm{eff}}v^{2}=0$$

From Equation (18), we can calculate the horizontal speed as:

(19) $$v_{\mathrm{hor}}=\sqrt{\frac{2\sqrt{1-{\left(\frac{m_{\mathrm{tot}}g}{T}\right)}^{2}}\cdot T}{\rho c_{D}A_{\mathrm{eff}}}}$$

By using the thrust-weight ratio *TWR* and substituting *T* = *TWR*⋅*m_tot_*⋅*g* we get

(20) $$v_{hor}=\sqrt[{4}]{1-\frac{1}{TWR^{2}}}\sqrt{\frac{2m_{tot}g}{\rho c_{D}A_{eff}}}\sqrt{TWR}$$

The first square root requires for the *TWR* to be at least 1.3. The effective area $A_{\mathrm{eff}}$ is a function of the forward pitch angle *α*. To simplify the calculation we only take into account the vertical projection of the quadcopter top area:

(21) $$\sin\alpha =A_{eff}/A=m_{tot}g/T=1/TWR$$

and as a result we get:

(22) $$v_{hor}=\sqrt[{4}]{1-\frac{1}{TWR^{2}}}\sqrt{2\frac{T}{\rho c_{D}A}}\sqrt{TWR}$$

Equation (22) becomes:

(23) $$v_{hor}=\sqrt[{4}]{1-\frac{1}{TWR^{2}}}\sqrt{\frac{2m_{tot}g}{\rho c_{D}A}}TWR$$

The top area *A* can be calculated also taking into account the motor-to-motor distance (*MTM*) and the propeller size ($r_{prop}$):

(24) $$A=\frac{1}{2}\;MTM^{2}+3\cdot \pi r_{prop}^{2}=\frac{1}{2}{\left(0.16\right)}^{2}+3\pi {\left(0.046\right)}^{2}=0.033\;m^{2}$$

The power consumption for propulsion will be:

(25) $$p_{prop}=Tv_{hor}=T\sqrt[{4}]{1-\frac{1}{TWR^{2}}}\sqrt{\frac{2m_{tot}g}{\rho c_{D}A}}TWR$$

For longer MD, it is necessary to fly under ideal conditions (no wind and at a room temperature of 20 °C). However, the movement during the flight creates a fluid-air displacement of intensity equal to $v_{hor}$ but in the opposite direction ($v_{air}=v_{hor}$) therefore, the power is obtained by assuming that the body drag ($F_{D}$) is proportional to airspeed ($v_{\mathrm{air}}$):

(26) $$p_{prop}=F_{D}\cdot v_{air}=\frac{1}{2}\rho C_{D}Av_{air}^{3}$$

**Figure 4:**
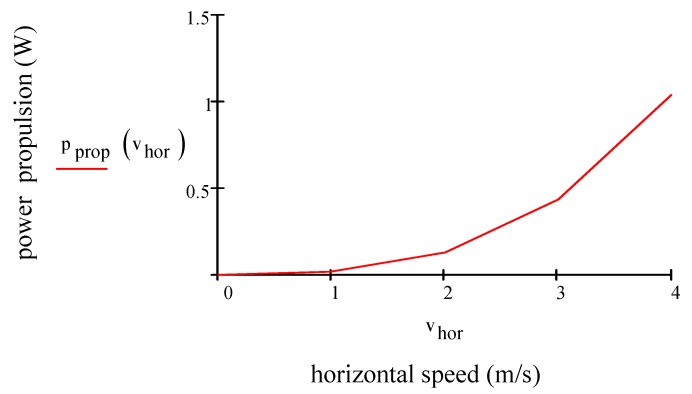
Electric power consumption for one motor vs. horizontal speed.

By considering Equations (22)–(26), the results are $v_{hor}=$ 4.20 m/s, $p_{prop}=$ 4.80 W (calculated for four motors), $p_{av}=$ 1.57 W, $p_{pld}=$ 1.21 W, $p_{tot}=p_{prop}+p_{pld}+p_{av}=$ 7.57 W. By using Equation (9) $t_{hor}=$ 9.38 min. Figure 4 plots the electric power consumption of the propulsion motor (W) vs. horizontal speed (m/s) for one motor. Electric power consumption, which is calculated for one motor increases slightly and exponentially when the drone flies along the horizontal direction.

#### 2.2.4. Impact of Weights

The MAV owes its success to its ability to carry a payload. However, the weight of a payload limits the mission parameters in particular travel distances. We observe that power consumption increases almost linearly when the weight of a payload increases. The maximum weight of a payload depends on the thrusts that the motors can produce. By considering a payload of 6 g, Equation (26) shows that $p_{prop}=$ 4.80 W and $p_{tot}=$ 7.57 W. Equation (27) shows, known the motor efficiency and specific energy of the battery, how the payload’s weight could affect the travel distance of a MAV. D’Angelo estimates the energy requirement to be [18,22,23,24,25]:

(27) $$\frac{d}{1-v_{r}}\left(\frac{m_{v}+m_{p}}{370\eta r}+\frac{p_{tot}}{v_{c}}\right)$$

**Figure 5:**
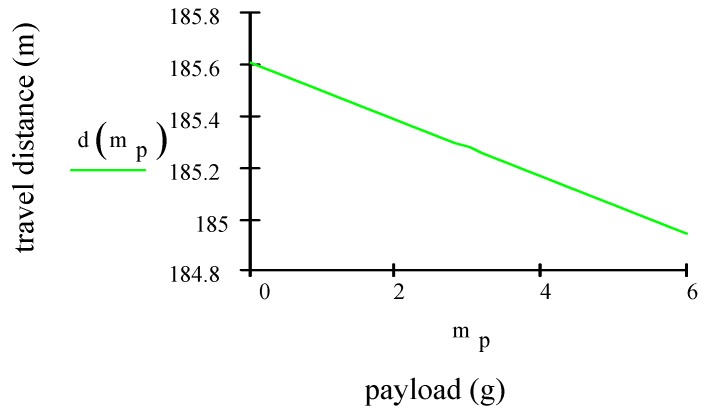
Travel distance vs. payload.

The travel distance *d* in meters is:

(28) $$d=\frac{2\cdot E_{b}(1-v_{r})}{\left(\frac{m_{p}+m_{v}}{370\eta r}+\frac{p_{tot}}{v_{c}}\right)}$$

where $E_{b}$ is the source specific energy of the battery (for Li-Po 100–265 Wh/kg), $m_{p}$ = payload mass (grams), $m_{v}$ = vehicle mass (g), *r* = lift-to-drag ratio, η = power transfer efficiency for motor and propeller, $p_{tot}$ = power consumption of MAV in W, $v_{r}$ = ratio of headwind to airspeed and $v_{c}$ = cruising velocity of the aircraft in m/s. The values used in Equation (28), to calculate the travel distance d are $E_{b}$ = 100–265 Wh/kg, $v_{r}=$ 0.3, $m_{p}=$ 6 g, $m_{v}=$ 74 g, η=0.8, r=3, $p_{tot}=$ 7.57 W, $v_{c}=$ 4.20 (m/s). Figure 5 plots the travel distance vs. payload. The aircraft’s travel distance is roughly proportional to the payload mass. As payload mass increases, the total weight of the aircraft increases. Increasing the weight raises the minimum speed of the aircraft and reduces the travel distance.

**Figure 6:**
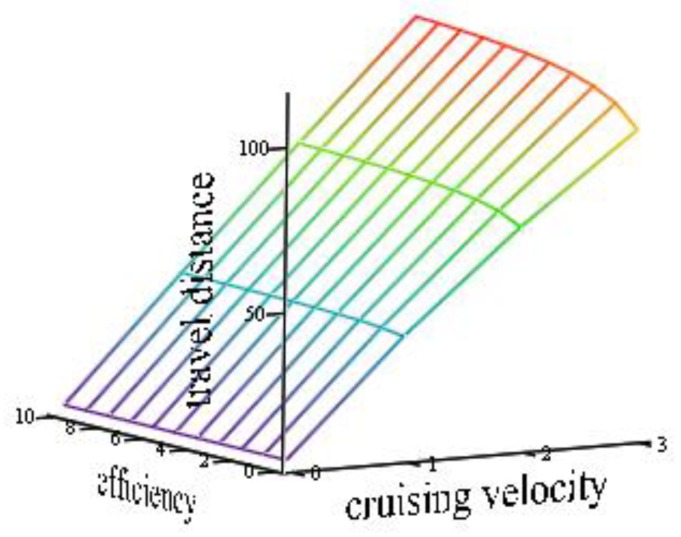
Travel distance as a function of motor efficiency and cruising velocity.

Figure 6 plots the travel distance as a function of cruising velocity and motor efficiency. The variation of motor efficiency with cruising velocity leads to increased travel distance. This means that power will increase because the motor will have to spin faster and absorb more current.

#### 2.2.5. Impact of Wind

Wind condition is a major environmental factor that affects the power consumption of a MAV. We are only considering the flight of a MAV in headwind conditions. In this scenario, the motors are forced to generate more thrust to maintain a horizontal condition and thrust requires power. The drone was set to fly with a wind speed of 7 m/s. Equation (25) will be re-written considering the power needed to overcome $F_{D}$ in headwind conditions [18,22,23,24,25]:

(29) $$p_{prop}=F_{D}\cdot v_{wind}=\frac{1}{2}\rho C_{D}Av_{wind}^{3}$$

By considering Equation (29), we have the results $v_{wind}=$ 7 m/s, $p_{prop}=$ 22 W (calculated for four motors), $p_{av}=$ 1.57 W, $p_{pld}=$ 1.21 W, $p_{tot}=p_{prop}+p_{pld}+p_{av}=$ 24.78 W. By using Equation (9) $t_{wind}=$ 3.17 min.

**Figure 7:**
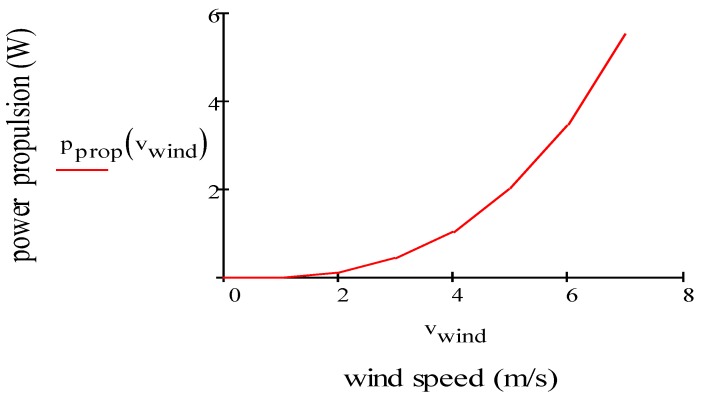
Power consumption of propulsion for one motor vs. wind speed.

Figure 7 plots the electric power consumption of the propulsion motor (W) vs. wind speed (m/s) for one motor. Electric power consumption calculated for one motor increases exponentially when the drone flies along the horizontal direction in headwind condition. In fact, in this condition, the motor will have to spin faster and absorb more current.

**Figure 8:**
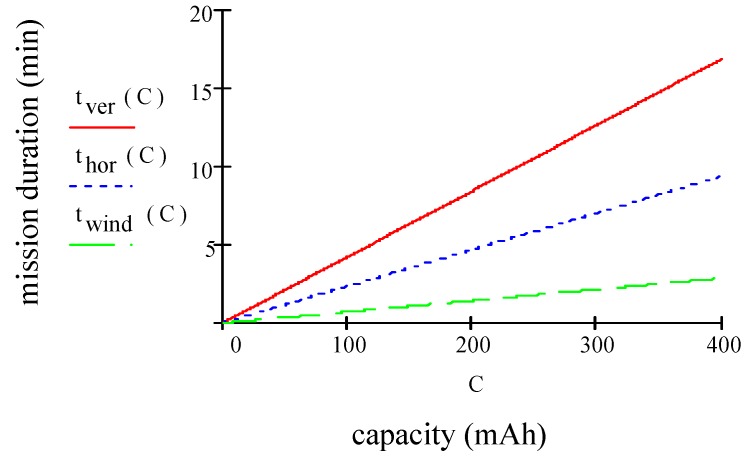
Mission duration vs capacity.

Figure 8 plots MD in vertical, horizontal and in headwind condition. In these conditions, MD increases as a function of the battery capacity. However, as the capacity of battery extends, the increase in MD becomes ineffective.

## 3. Economical Alternative to PV

This section describes the design and simulations of a novel harvester based on plasmonics technology, which is able to improve mission parameters. Moreover, to optimize the power transfer, an optimal matching between harvester and load is proposed.

### 3.1. Novel Harvester to Power Small Aerial Vehicles

**Figure 9:**
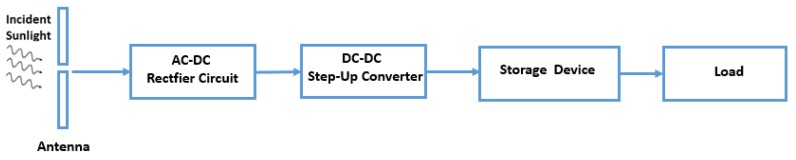
Block diagram of an energy harvesting system.

The model presented above shows that the weight of the battery plays one of the most important roles in determining the mission parameters of a flying object. Energy harvesting gives the opportunity of reducing dimensions and weight of the battery reducing the total weight of a MAV and consequently decreasing the amount of energy required to power it. This harvester is called nano-rectenna. The term results from joining “rectifying circuit” and “nano-antenna” [17], i.e., a novel answer to the power issues of the MAVs. Figure 9 shows the block diagram of the energy harvesting system proposed.

Each rectenna contains a receiving antenna, a rectifier circuit, a DC-DC step-up converter, a storage device and a load as shown in Figure 9. These nano-antennas use excited localized surface plasmons to induce electrons flow along the antenna, generating alternating current (AC) at the same frequency of the incident wave [17]. Subsequently, the rectifying circuit receives the AC from the dipole metallic strips. After the rectifier, the output presents a unidirectional current flow and a not constant voltage. To make this output voltage constant, a capacitor is usually added at the rectifier’s output. The battery and load need to be fed at a specific and regulated voltage value. A DC-DC boost converter is introduced after the rectifier, so a low output voltage of the rectenna array can be increased to a typical battery voltage. After the DC-DC block, the energy is then stored in the energy storage device, often chosen as a rechargeable battery/supercapacitor. These flexible dipole rectennas are thought to be incorporated into the frame of a MAV [26]. In the following paragraph, the complete characterization of a rectenna in Figure 9 is presented.

### 3.2. Internal Nano-Antenna Impedance Evaluation

**Figure 10:**
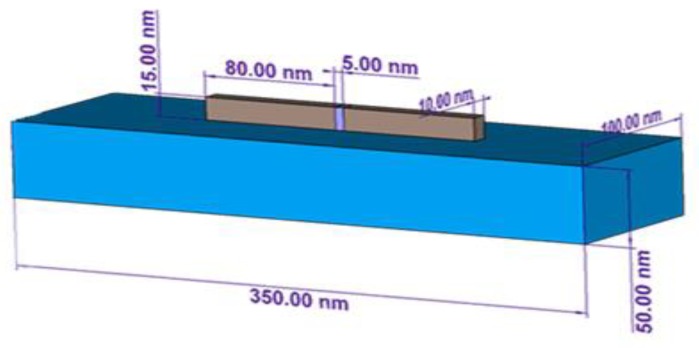
Schematic representation of the aluminum nano-dipole antenna, defining its geometrical parameters.

The antenna operating in receiving mode can be represented by a voltage source $V_{open}$ and an input impedance in series $Z_{A}=R_{in}+jX_{in}$ · $V_{open}$ represents the open circuit voltage at the terminals of the antenna when the load is not connected. $Z_{A}$ represents the impedance of the antenna characterized by a resistive component $R_{in}$ defined as the sum of the radiation resistance $R_{rad}$ and loss resistance $R_{loss}$ and by a reactive component $X_{in}$ (that is equal to zero at resonant frequency). In order to evaluate the nano-antenna impedance, the study has been carried out focusing on aluminum [27] nano-dipole antenna shown in Figure 10.

Aluminum is a low-loss metal whose permittivity at the frequency of interest is $\varepsilon (\omega )=\varepsilon^{\prime }(\omega )+i\varepsilon^{\prime \prime }(\omega )=$ −90.56 + i38.56 where $\varepsilon^{\prime }(\omega )$ and $\varepsilon^{\prime \prime }(\omega )$ respectively represent real and imaginary parts of the complex permittivity ε(ω) [14,28]. The numerical analysis in this section was performed with a commercial full-wave 3D electromagnetic simulator CST Studio Suite 2016.

**Figure 11:**
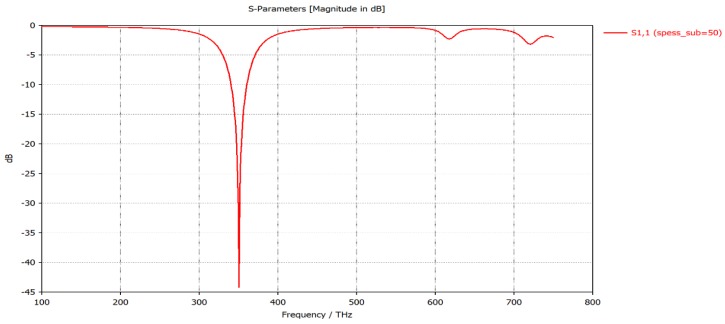
S-parameter magnitude (dB) vs. Frequency.

The dipole consists of two identical arms of length *L*. At 350 THz ( λ= 857 nm) we set antenna’s length *L* as $L=\frac{\lambda_{eff}}{2}=\frac{\frac{\lambda }{2}}{n_{eff}}=80$ nm, where $n_{eff}$ = 2.8, numerically extracted for λ= 857 nm from [28] represents the effective refractive index, $\lambda_{eff}$ represents the effective wavelength experienced by the plasmonic dipole, which is shorter than the free-space wavelength $\lambda_{0}$. The width and the height of the arms are fixed respectively at 10 nm and at 15 nm. The gap G between the two arms is fixed at 5 nm. The substrate on which the aluminum nano-antennas are patterned is assumed to be a silicon dioxide (SiO_2_) (the substrate thickness is fixed at 50 nm, length and width are fixed respectively at 350 nm and 100 nm, the related dielectric permittivity is 4.82 and the dielectric loss tangent is 0.002). The feeding point at the center of the antenna is set to a lumped port instead of a real diode for simplicity [29,30]. In general, the maximum transferred energy obtained when the antenna is resonant is measured at the minimum of the return loss (S_11_) parameter shown in Figure 11. The antenna is resonating at 350 THz with a return loss of −45 dB. At this value of return loss, the maximum power transmitted to the antenna is 99.87% [15].

**Figure 12:**
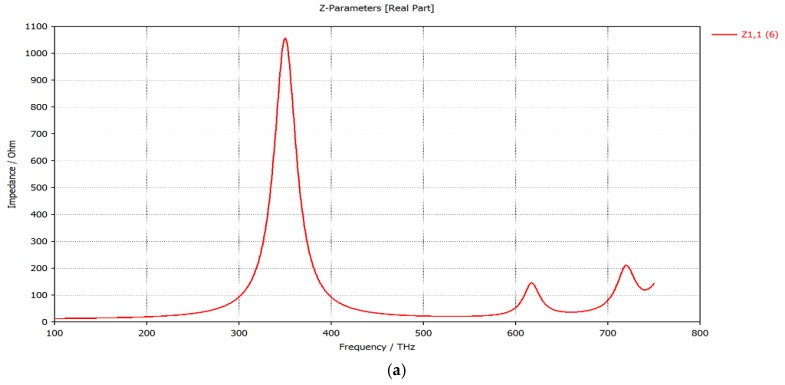

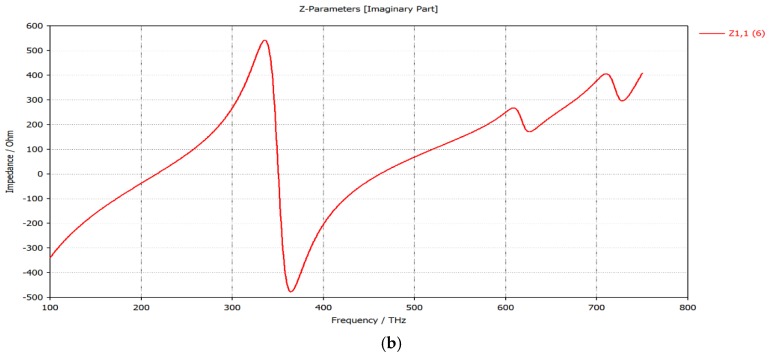
(**a**) Input resistance of the proposed antenna; (**b**) Input Reactance of the proposed antenna.

To determine the input impedance, the gap is excited by a default Gaussian pulse at the frequency of 350 THz. Figure 12 shows input impedance of a dipole antenna in resonance condition: (a) input resistance $R_{in}$ = 1055 Ω and (b) input reactance $X_{in}$ = 0 Ω.

### 3.3. Effective Area Evaluation

A parameter usually used in the antenna theory is the effective area $A_{eff}$ of the antenna calculated by Equation (30) [16]:

(30) $$Aeff=\frac{V_{open}^{2}Z_{0}}{4E_{i}^{2}Z_{A}}$$

**Figure 13:**
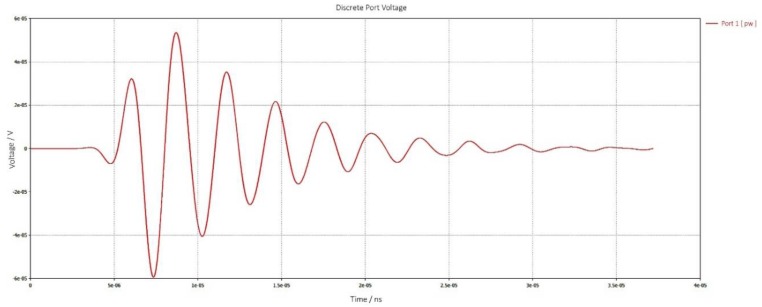
Open circuit voltage of the dipole when the load is not connected.

During the simulation, the nano-dipole was irradiated by a linearly polarized plane wave at a frequency value of 350 THz with an arbitrary amplitude of 1 V/m. By assuming, for $V_{open}$ the simulated value of 5 × $10^{-5}\;$ V as reported in Figure 13, calculated when the load is not connected, for the intrinsic impedance of free space $Z_{0}$ a value of 377 Ω, for the nano-dipole impedance $Z_{A}$ a value of 1055 Ω and for the incident electric field $E_{i}$ a value of 1 V/m, the value of nano-dipole effective area $A_{eff}$ calculated by (30) is 223 nm².

### 3.4. Electrical Parameters Evaluation

The energy radiated by the Sun with a temperature equal to 5778 K follows the Plank’s blackbody radiation formula [16]:

(31) $$W_{\lambda }=\frac{2\pi hc^{2}}{\lambda^{5}(e^{\frac{hc}{\lambda kT}}-1)}$$

where *h* is the Plank’s constant equal to $6.63\;\times 10^{-34}$ [J s], c is the speed of light equal to 3·10^8^ [m/s], T is the temperature [k], and K is the Boltzmann’s constant equal to $1.38\;\times 10^{-23}$ [J/K]. To obtain the power density (Poynting vector) on the antenna we are integrating $W_{\lambda }$ between $\lambda_{\min}$ and $\lambda_{\max}$:

(32) $$S=\int_{\lambda \min}^{\lambda \max}W_{\lambda }(\lambda ,T)d\lambda$$

In this case, the actual Poynting vector has been obtained by taking into account the radiation efficiency $\eta^{rad}$ whose values depend on the wavelength and by integrating $W_{\lambda }$ in the operating range of the optical nano-antenna, i.e., between $\lambda_{1}=300$ nm and $\lambda_{2}=1200$ nm [31,32,33]. This range of wavelengths is commonly used in literature because it covers the visible and infrared interval. Equation (32) can be rewritten as:

(33) $$S^{\prime }=\int_{\lambda_{1}}^{\lambda_{2}}W_{\lambda }(\lambda ,T)\eta^{rad}(\lambda )d\lambda$$

The available power on the load matching condition has been evaluated as:

(34) $$P=A_{eff}\cdot S^{\prime }$$

Table 2 shows the values of the actual Poynting vector and the corresponding power on the load in matching conditions by considering a dipole with arm length of 80 nm.

**Figure 14:**
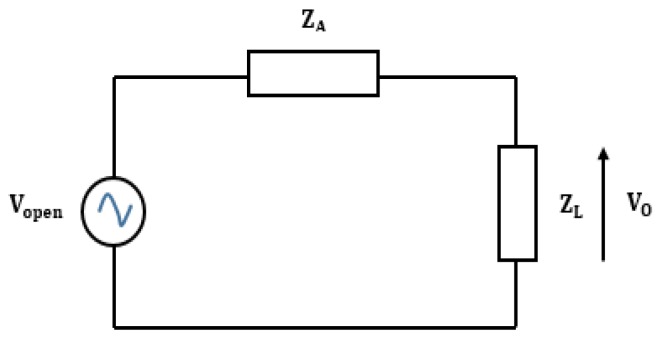
Equivalent circuit for the calculation of the output voltage $V_{0}$.

The maximum value of the electrical field has been calculated as:

(35) $$E_{i}=\sqrt{2\cdot \left|\langle S^{\prime }\rangle \right|\cdot \sqrt{\frac{\mu_{o}}{\varepsilon_{o}}}}$$

where $\sqrt{\frac{\mu_{o}}{\varepsilon_{o}}}$ is the intrinsic impedance of free space $Z_{0}$ equal to 377 Ω. Figure 14 shows the equivalent circuit, which serves to calculate the output voltage $V_{0}$ obtained when the load is connected to the nano-antenna [16]

Assuming the impedance matching condition between the antenna $Z_{A}$ and the load $Z_{L}$, the output voltage $V_{0}$ is equal to $\frac{V_{open}}{2}$. Table 3 shows the values of the incident electric field, of $V_{open}$ and of the output voltage $V_{0}$ for dipole arm length of 80 nm.

Unfortunately, the results above show that the output power and voltage of a single rectenna tuned at 350 THz are very low, of the order of nW and μV, respectively. These values are limited to the characteristics of the metal insulator metal diode (MIM) or multi insulator metal diode (MIIM) [11,34]. For aeronautic applications, a MAV requires high current and low voltage. Consequently, an arrangement in array is necessary. The dipole rectenna elements are connected in series and parallel to meet the requirement of increased power. To obtain an efficient power transfer, the coupling between the impedance of the array and the impedance of the DC-DC boost power converter is required. Therefore, the array resistance has to be sufficiently low and close to the DC-DC resistance. Consequently, a reduction in power loss is achieved.

### 3.5. Design of a Rectenna Array

#### 3.5.1. Impedance Matching

The rectenna model used in this paper is reported in [3]. To calculate the impedance matching supposed above, the diode junction capacitance $C_{D}$ can be neglected because it behaves as a low pass filter and only the DC component can be considered. The current flowing in the circuit can be expressed as [16]:

(36) $$I=\frac{Vopen}{RA+\frac{RDRL}{RD+RL}}=Vopen\cdot \frac{RD+RL}{RA\left(RD+RL\right)+RDRL}$$

The total power in the circuit can be expressed as:

(37) $$PTOT=Vopen^{2}\cdot \left[\frac{RD+RL}{RA\left(RD+RL\right)+RDRL}\right]$$

The power in the antenna, in the diode and in the load are shown below:

(38) $$PA=Vopen^{2}\cdot {\left[\frac{RD+RL}{RA\left(RD+RL\right)+RDRL}\right]}^{2}\cdot RA$$

(39) $$PD=Vopen^{2}\cdot {\left[\frac{RL}{RA\left(RD+RL\right)+RDRL}\right]}^{2}\cdot RD$$

(40) $$PL=Vopen^{2}\cdot {\left[\frac{RD}{RA\left(RD+RL\right)+RDRL}\right]}^{2}\cdot RL$$

The best impedance matching condition is obtained when the value of RD is much larger than the values of RA and RL, on the contrary, RA and RL must be equal. Therefore, the optimal matching can be achieved by a rectenna array whose equivalent impedance equals the impedance $Z_{\mathrm{Iboost}}$ of the DC-DC boost converter.

#### 3.5.2. DC-DC Boost Power Converter

Figure 9 shows a DC-DC boost converter introduced after the rectifier, so a low output voltage of the rectenna array can be increased to a typical battery voltage [35,36,37]. By following this approach, an LTC3108 DC/DC boost converter, is used [38]. LTC3108 produces an output voltage up to 5 V with a peak current of 4500 μA under 500 mV input voltage and a resistance of 3 Ω as input conditions. The knowledge of the DC-DC impedance (3 Ω) is important for obtaining the optimal matching with that of rectenna array.

#### 3.5.3. The Equivalent Circuit of an Array of Optical Rectennas

**Figure 15:**
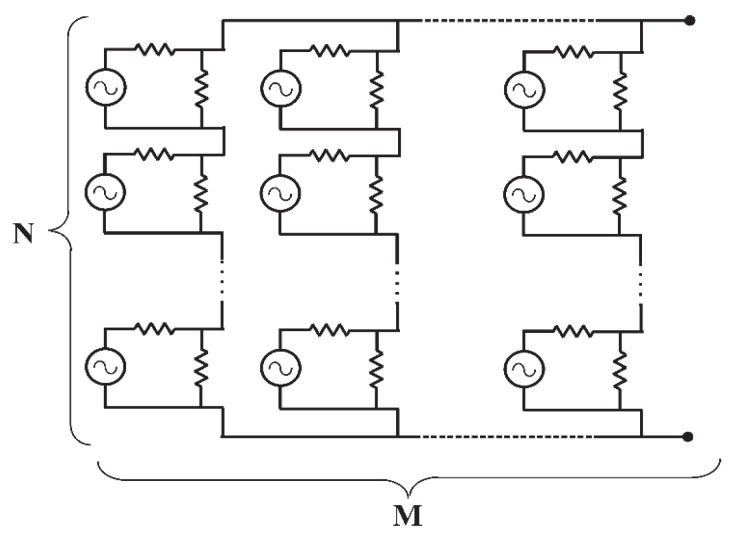
Equivalent circuit of an array of N·M optical rectennas, each cell corresponds to the circuit in Figure 14.

The equivalent circuit of an array of optical rectennas is shown in Figure 15. Each cell consists of an antenna with the rectifier placed in the gap. The equivalent resistance of a single rectenna *R_RECT_* can be expressed as [16]:

(41) $$R_{RECT}=\frac{R_{A}R_{D}}{R_{A}+R_{D}}$$

Currently, the state of the art indicates a diode resistance $R_{D}$ around 1.2 MΩ [39], whereas the value of $R_{A}$ is equals to 1055 Ω. If N rectennas are connected in series (matrix column), the value of the column resistance $R_{COL}$ becomes equal to:

(42) $$R_{COL}=N\cdot R_{RECT}=N\cdot \frac{R_{A}R_{D}}{R_{A}+R_{D}}$$

$R_{COL}$ is higher than the DC-DC resistance, therefore the impedance matching between the array and the DC-DC boost converter is not satisfied. To obtain the maximum power transfer between the rectennas array and the DC-DC converter, it is necessary to decrease $R_{COL}$ connecting in parallel M column. Therefore, the equivalent resistance of an array is given by:

(43) $$R_{eq,\;array}=\frac{N}{M}\cdot R_{RECT}=\frac{N}{M}\cdot \frac{R_{A}R_{D}}{R_{A}+R_{D}}$$

and the ratio of N/M is obtained as:

(44) $$\frac{N}{M}=R_{eq,\;array}\cdot \left(\frac{R_{A}+R_{D}}{R_{A}\cdot R_{D}}\right)$$

With this method, the series connection, allows the open circuit voltage to be increased whereas the parallel connection gives the degree of freedom needed to achieve the impedance matching with the load. In a matrix arrangement, the dipoles are considered as simply connected in series-parallel, however, the practical realization of this matrix would require a further study to avoid parasitic interactions among them; this aspect will be investigated in the future. To achieve the voltage matching between the optical rectenna array and the DC-DC converter, the rectenna array output voltage has to be greater than the lower input voltage of the DC-DC converter. For this case study, the maximum output voltage of the array $V_{0,array}$ is fixed at 1000 mV (this value will decrease to 500 mV when the matching load is connected to the array). N can be obtained as [16]:

(45) $$N=\frac{V_{O,\;array}}{V_{DC}}=\frac{2V_{Iboost}}{\frac{V_{open}}{\pi }}$$

where the DC value can be obtained from the $V_{open}$ multiplied for $\frac{1}{\pi }$ (which corresponds to an half-wave rectifier). From Equation (45), it is possible to calculate the value of *M*:

(46) $$M=\frac{N}{R_{eq,\;array}\cdot \left(\frac{R_{A}+R_{D}}{R_{A}\cdot R_{D}}\right)}$$

The array area can be expressed as:

(47) Aarray=N⋅M⋅Adipole

Table 4 shows the values of N and M for a nano-dipole which arm length is of 80 nm.

By considering Equation (47), the rectenna array area, constituted by dipoles, which arm length, is of 80 nm is 23 cm².

#### 3.5.4. Load Power and Energy Evaluation

**Figure 16:**
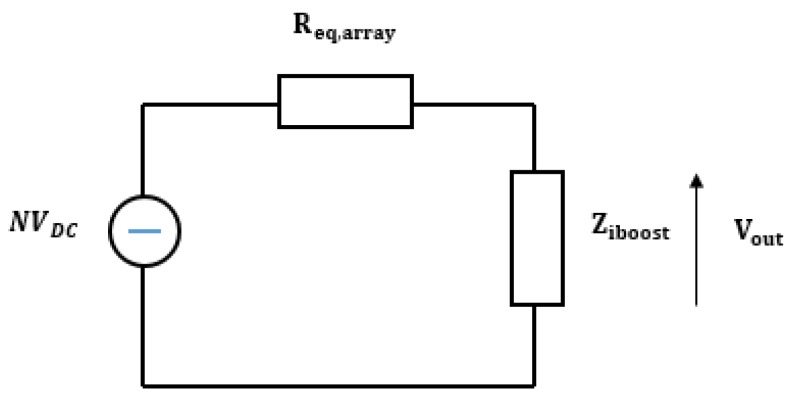
Circuit for the calculation of a rectenna array power.

In order to evaluate the available power on the load, the whole array of rectennas can be simulated as the equivalent circuit shown in Figure 16 [16].

**Figure 17:**
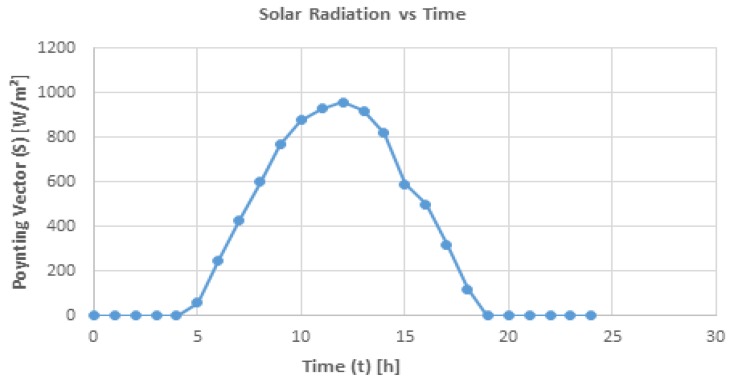
Solar radiation versus time.

The impedance matching condition between the rectenna array $R_{eq,array}$ and the load $Z_{iboost}$ has been assumed. The input impedance $Z_{iboost}$ is considerate equal to 3 Ω. The load power can be calculated by:

(48) $$P_{load}=\frac{V_{out}^{2}}{Z_{Iboost}}$$

where the output voltage $V_{out}$ on the load $Z_{iboost}$ is equal to $N\frac{V_{DC}}{2}$. Under matching impedance conditions, the array output power and the boost input power are the same and equal to 84.14 mW. Figure 17 shows the curve of solar radiation versus time during a typical July day in a southern Italian region. It should be noted that the maximum of the solar radiation is at the Zenith. The energy that can be delivered by a rectenna array can be expressed as:

(49) $$\xi_{day}=\int_{t}P_{array}\frac{R(t)}{R_{\max}}dt$$

where R(t) is the solar radiation versus time, $R_{\max}$ is the value of solar radiation at Zenith and $P_{array}$ is the maximum value of the array power. By considering Equation (49), the obtained energy is equal to 2572 J. By taking into account the above results, Equation (9) shows improvements for the mission parameters.

For MD the improvements with the harvester only in horizontal condition has been of 16.30 mins. With a hybrid system (battery and harvester), the improvements in horizontal and headwind conditions have been respectively of 50 min and 15.30 min. For travel distance, the improvements with the harvester only have been of 1903 m and with a hybrid system of 5845 m. In headwind condition, considering the hybrid system, the improvement has been of 3047 m. The preliminary results above show that a dipole rectenna array with 22,066,058 elements in parallel and 62,800 elements in serial connections are sufficient to recharge a small battery on board a MAV. Finally, the angle of incidence of solar radiation on a dipole rectenna array embedded into the frame of a MAV may vary with the instantaneous change in orientation of a flying MAV. When the pole orientation of rectennas does not match with the electric field of the incident solar radiation, the rectenna may not generate a high output power. To overcome this polarization problem, a polarization-free dipole rectenna array configuration with six difference polarity directions is mandatory. For this application, the state of the art recommends a typical arranging of the dipole rectenna elements in a circle [40,41,42].

## 4. Energy Storage

Figure 9 shows the working principle of the novel harvester. From this figure, it is possible to see that the harvester does not feed the load directly but recharges a small battery. In fact, the harvester has an intermittent nature, which makes the voltage and current unstable. On the other hand, the battery output can supply the load with a stable voltage and current. For EH application is imperative to use rechargeable batteries. 

In this study, a Li-Po ultra-fast rechargeable battery with 3.7 V nominal voltage and a capacity of just 400 mAh is used, taking into account the tradeoff between battery life and its size. To extend the mission parameters, Equation (50) explains, as at any instant t, the available energy must be greater or equal to the required energy for supporting the load:

(50) $$E_{harvested}(t)+E_{stored}(t)\geq E_{load}(t)+E_{loss}(t)$$

where $E_{harvested}$ is the generated energy at any instant *t* by optical dipole rectenna array, $E_{stored}$ is the energy stored in a storage device (battery), $E_{load}$ is the energy required from the load and $E_{loss}$ is the energy dissipated due to the Joule effect. The maximum charge current rate fixed from the DC-DC boost converter is 4500 μA. During the normal operation, a Li-Po battery is discharged only for 20% of its capacity, therefore the recharging time is of 2.5 h. At this state, it is easy to recharge the battery with the power delivered by the energy harvester, rather than charging the battery at a later stage, when it is fully discharged. This preliminary study shows that the current flow of this energy harvester is not enough to fastly charge the battery while the MAV is mid-air, however, in the near future, a hybrid system constituted by this energy harvester and a novel battery able to be recharged during the flight, in less than 1 min, could power the system indefinitely [43,44,45].

## 5. Conclusions

In this paper, a possible solution on how to improve mission parameters (MD and travel distance), for a micro air vehicle (MAV) has been given. This paper has been divided into two different parts. Initially, we have proposed a model that identifies all factors that determine the power consumption of a drone. The results of the MD for several scenarios proposed shown that in hovering condition MD is 18.16 min. In vertical conditions, MD is only 17.20 min. In horizontal condition, MD is only 9.38 min. Finally, in worst-case condition, i.e., in headwind conditions, MD is only 3.17 min. MD and travel distance are limited by the capacity of the energy storage system. In the second part, to answer on how to improve mission parameters, we have designed and simulated a harvester using a flexible optical dipole rectenna array tuned at a frequency of 350 THz thought to be implemented into the frame of a MAV. Moreover, a novel method to make the impedance of the harvester match that of load has been proposed. In light of the results above, Equation (9) shows improvements for the mission parameters. For MD, the improvements with the harvester only in horizontal condition have been of 16.30 min. With hybrid system (battery and harvester), the improvements in horizontal and headwind conditions have been respectively of 50 min and 15.30 min. For travel distance, the improvements with harvester only has been of 1903 m and with hybrid system of 5845 m. In headwind condition, by taking into account the hybrid system, the improvement has been of 3047 m. This is in line with theoretical and simulations results because the power captured by the nano-antennas is largely lost on the rectifier. Finally, in the next future, a hybrid system constituted by this harvester and a novel battery able to be recharged during the flight in less than one-minute could power the system indefinitely. In light of the considerations, which were obtained from the results discussed above, we can assert that nano-antennas, although still at the initial stage and not of immediate application, represent a new, stimulating and not yet consolidated topic, which will be able to foster new research activities in the aeronautic field.

## Figures and Tables

**Table 1:** Estimated weights of propulsion, payload and avionics components.

Parameter	Value	Description
*m_prop_*	24.4 (g)	Propulsion mass
*m_payl_*	6.3 (g)	Payload mass
*m_av_*	23 (g)	Avionics mass (including all cabling)
*m_airframe_*	10 (g)	Airframe mass
*m_array_*	2 (g)	Dipole rectenna array mass
*W_tot_*	80 (g)	Total Weight MAV

**Table 2:** Poynting Vector and Optimal Power.

L [nm]	Actual Poynting Vector <S’> [W/m^2^]	Power P [nW]
80	70	15.6

**Table 3:** Electric Field, V_open_ and Output Voltage V_0._

L [nm]	E_i_ [V/m]	V_open_ [µV]	V_o_ [µV]
80	230	50	25

**Table 4:** Parameters of a Rectennas Array.

L [nm]	Z_Iboost_ [Ω]	V_DC_ [µV]	V_o,array_ [mV]	N	M
80	3	16	1000	62,800	22,066,058

## Abbreviations

The following abbreviations are used in this manuscript:

UAVs	Unmanned Aerial Vehicles
MAVs	Micro Air Vehicles
MD	Mission Duration
EH	Energy Harvesting
SWIR	Shortwave Infrared
PV	Photovoltaic
TWR	Thrust to Weight Ratio
AC	Alternating Current
DC	Direct Current
MIM	Metal Insulator Metal diode
MIIM	Multi Insulator Metal diode
